# Lessons from an Adaptation of A Technology‐based Platform to Assist Dementia Caregivers in Rural Michigan

**DOI:** 10.1002/alz70858_105343

**Published:** 2025-12-26

**Authors:** Daniel Velez Ortiz, Fei Sun, Cheryl Hassen‐Swarthout, Arienne Patano

**Affiliations:** ^1^ Michigan State University, East Lansing, MI, USA

## Abstract

**Background:**

Alzheimer's disease and related dementias (ADRD) affect approximately 202,800 residents in Michigan, with many living in rural areas. These conditions impose significant financial, physical, and psychological burdens on family caregivers, who play a critical role in supporting this population. Providing access to resources and support is crucial to dementia care, as there is currently no medical cure for ADRD. However, rural caregivers face persistent challenges, including limited availability of services, transportation barriers, insufficient knowledge of ADRD and care, and a lack of culturally appropriate services.

**Aims:**

This project seeks to address disparities in access to dementia support and services for underserved rural communities by providing a technology‐based platform. A particular focus is placed on rural communities, with an emphasis caregivers.

**Approach:**

The project employs a two‐tiered, technology‐assisted service model developed through partnerships between the MSU School of Social Work and the Region 9 Area Agency on Aging. Tier 1 – Self‐Paced Support: Delivered through the Trualta online platform, this level provides users with information, training, and tailored dementia‐related resources. Tier 2 – Staff‐facilitated Support: Offered by MSW staff and interns, this includes both in‐person and online dementia education and caregiver support services. This tier also enhances the workforce capacity of staff and interns to assist families affected by dementia, particularly in rural and minority communities.

**Findings:**

About 40% of caregivers registered on the Trualta platform are over 60, with slightly less than 40% aged 46–60. Approximately 14% of users are male. The overall use level is moderate. The most frequently accessed resources include caregiver wellness, balancing work and caregiving, and practical tips for toileting and transferring. Other popular features include monthly seminars and community forums. Personalized training was well received among rural participants and showed improve knowledge of ADRD and reduction of caregiver stress.

Implications in terms of improving access to dementia services in rural communities reflect on the sustainability of the community collaboration model, highlighting its potential to enhance the quality of life for families affected by dementia across rural populations in Michigan.

